# The Obesity‐Related Indices Are Useful for Predicting Diabetic Nephropathy in Patients With Type II Diabetes Mellitus: A Retrospective Cohort Study of NHANES

**DOI:** 10.1002/edm2.70087

**Published:** 2025-08-08

**Authors:** Wei Zhang, Yao Liu

**Affiliations:** ^1^ Xi'an International Medical Center Hospital Xi'an People's Republic of China; ^2^ Xi'an XD Group Hospital Xi'an People's Republic of China

## Abstract

**Background and Aim:**

This study investigated the correlation between obesity‐related indices and diabetic nephropathy (DN) in patients with type II diabetes mellitus (T2DM). It aimed to provide a new predictive assessment tool for the clinic and a scientific basis for managing DN.

**Methods:**

The data utilised in this study were obtained from the National Health and Nutrition Examination Survey (NHANES) database, spanning 2007 to 2018. A total of 843 patients diagnosed with DN were included in the analysis. The association between obesity‐related indices and DN was investigated using multivariable logistic regression models. These relationships were further validated by restricted cubic spline (RCS) modelling.

**Results:**

The multivariable logistic regression analysis indicated that A Body Shape Index (ABSI), weight‐to‐waist index (WWI), body roundness index (BRI) and waist‐to‐height ratio (WHtR) levels were independently associated with DN. In particular, WWI, BRI, and WHtR levels were found to be independently associated factors with DN. Patients in the highest quartile of WWI, BRI, and WHtR exhibited a 1.19‐fold, 1.30 fold, and 1.30‐fold increased risk of DN, respectively, compared to patients in the lowest quartile. RCS analyses further confirmed the positive association between ABSI, WWI, BRI, and WHtR and DN. The ABSI offers incremental value in model predictive power for DN in patients with DM compared to other obesity‐related indices. As renal function deteriorates, BRI and WHtR show a significant negative correlation with estimated glomerular filtration rate (eGFR).

**Conclusion:**

Compared with other obesity‐related indices, the ABSI offers incremental value in model predictive power for DN in patients with DM.

## Graphical abstract

This study analysed NHANES data (2007–2018) to link obesity indices with diabetic nephropathy (DN) risk in type 2 diabetes. Multivariable regression and spline models identified ABSI, WWI, BRI, and WHtR as independent predictors. ABSI offered the best incremental predictive power for DN.

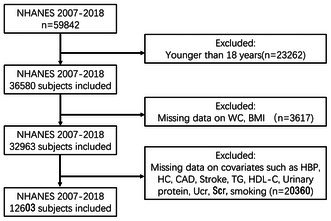

## 1 Introduction

DM is a chronic metabolic disease that poses a threat to global health, and its prevalence has been increasing annually due to changes in dietary habits and sedentary lifestyles [1]. DM‐related complications, especially diabetic microvascular complications (DMC) including cardiovascular disease and nephropathy, lead to poor prognosis and significantly reduce patients' quality of life [2]. DN is one of the most common microvascular complications of DM, characterised by glomerulosclerosis and tubulointerstitial fibrosis, ultimately resulting in chronic renal failure [3, 4]. DN is the primary cause of end‐stage renal disease (ESRD), which significantly increases patients' medical burden and risk of mortality [5]. The pathogenesis of DN is complex, involving multiple factors including glucose metabolism disorders, inflammation, oxidative stress, and other mechanisms [6, 7, 8]. More alarmingly, there is currently a lack of ideal early markers or indices to predict DN.

An accumulating body of research indicated obesity was particularly noteworthy as a prominent and influential risk factor contributing to the pathogenesis of DN [9]. Remarkably, the paradoxical protective association between overweight/obesity and DMC, a phenomenon well‐known as the obesity paradox, has been considered a non‐causal association based on methodological influences [10]. Dong Hoon Lee et al. reported individuals who were obese were at a heightened risk for developing DN due to the combined effects of suboptimal control of blood pressure and blood glucose levels [11]. However, other scholars revealed high levels of body fat variation rate due to weight loss were only associated with increased risk of diabetic DN in participants with BMI > 30 [12]. This contradictory result prompts us to delve deeper and find that the root cause may lie in the type of obesity and the specific fat distribution in DM patients that influence the incidence of DN. Different types of obesity, such as visceral obesity and subcutaneous obesity, exhibit significant differences in metabolic characteristics and their impact on organ function. Similarly, the fat distribution pattern in DM patients, particularly the accumulation of visceral fat, has been shown to be closely related to insulin resistance, chronic inflammation, and various metabolic disorders, all of which may directly or indirectly increase the risk of DN.

Therefore, to fully understand the relationship between obesity and DN, we must carefully distinguish between types of obesity and thoroughly analyse the individual fat distribution characteristics of DM patients. Consequently, a considerable number of researchers are interested in exploring the relationship between changes in obesity and DN by examining various obesity‐related indices such as body mass index (BMI), waist circumference (WC), waist‐to‐hip ratio (WHR), WHtR, lipid accumulation product (LAP), BRI [13], visceral adiposity index (VAI) [14], ABSI and WWI [15, 16, 17]. In addition, Cardiometabolic index (CMI) are lipid‐related parameters that reflect central obesity, which is closely associated with the development of DM [18]. However, the relationship between the aforementioned obesity‐related indices and DN remains unclear, and it is worth further investigating which indicator can better predict DN.

In the current study, we aimed to investigate the association of generalised and abdominal obesity, as measured by BMI, WC, WHR, WHtR, LAP, BRI, VAI, ABSI, WWI, and CMI, using data from the NHANES database, spanning the period from 2007 to 2018. The database offers a comprehensive repository of demographic, clinical, and biochemical data, providing a valuable resource for investigating DN and its associated risk factors. Following the implementation of rigorous cleaning and screening procedures, 843 eligible patients with DN were identified for analysis. The objective of this study was to comprehensively assess the relationship between obesity‐related indices and DN.

## 2 Methods

### 2.1 Study Population

The data utilised in this study were obtained from the NHANES database, spanning 2007 to 2018. This database contains the results of cross‐sectional surveys conducted every 2 years by the Centres for Disease Control and Prevention (CDC). The research protocol of the NHANES project adhered to the guidelines set forth by the Ethics Review Committee of the National Centre for Health Statistics (NCHS), and all participants were required to sign an informed consent form. In the course of data analysis, NIH policy regulations were adhered to. Given the anonymity and non‐direct contact nature of the data, it was used directly in the study without requiring additional ethical review. The study used the standards outlined in the Strengthening the Reporting of Observational Studies in Epidemiology (STROBE) statement to ensure the highest study design and reporting quality.

**FIGURE 1:**
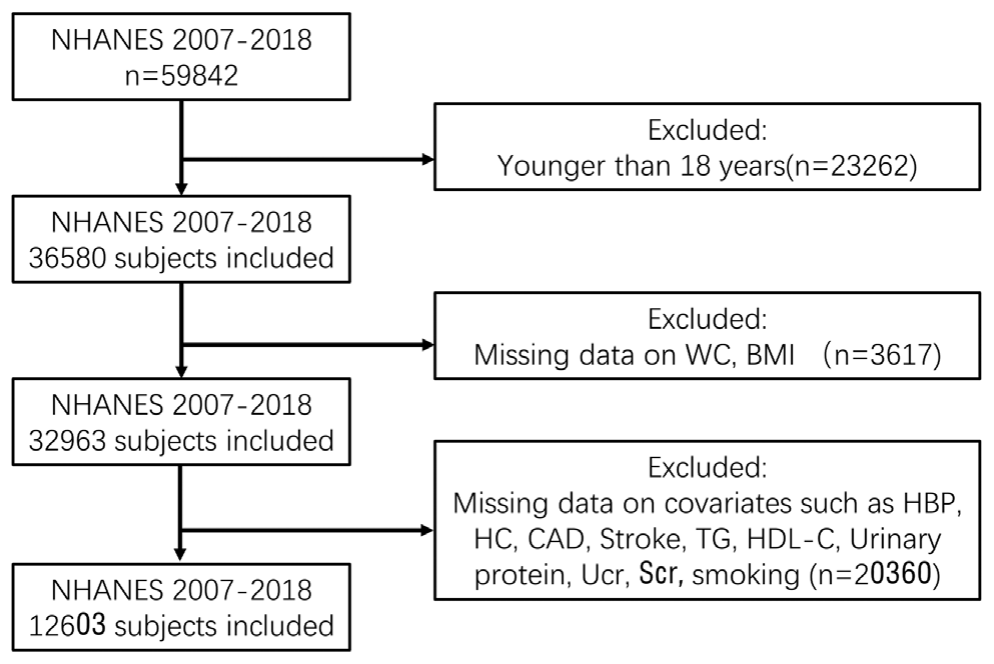
Flow chart of the participants.

At the study's outset, a sample population was selected from six consecutive survey cycles, comprising 12,603 participants. To guarantee the precision and relevance of the study outcomes, we rigorously implemented data cleaning and exclusion criteria to remove ineligible participants, including those under the age of 18 years (*n* = 23,262), individuals with missing data on WC, BMI (*n* = 3617), and patients with missing data on covariates such as Heparin‐Binding Protein (HBP), cancer, Coronary Artery Disease (CAD), stroke, Triglycerides (TG), High density lipoprotein cholesterol (HDL‐C), urinary protein, Urine Creatinine (Ucr), Serum Creatinine (Scr), and smoking (*n* = 20,360). Following the implementation of the aforementioned rigorous screening process, 12,603 eligible participants were identified for analysis in this study (Figure 1).

### 2.2 Definition of Disease

In this study, DM was defined according to one of the following criteria: (1) a definitive diagnosis by a qualified healthcare professional; (2) fasting plasma glucose (FPG) levels at or above the established threshold of 126 mg/dL; (3) glycosylated haemoglobin (HbA1c) levels of not less than 6.5%; (4) the individual's current use of diabetic medication or insulin therapy. To accurately assess renal function, we employed the urine albumin‐to‐creatinine ratio (UACR) and estimated glomerular filtration rate (eGFR) as the core indicators. The eGFR was calculated precisely according to the recommended formula by the Collaborative Group on Epidemiology of Chronic Kidney Disease (CKD‐EPI) [19]. The diagnosis of DN was based on internationally recognised criteria, namely a UACR value of at least 30 mg/g or an eGFR value of less than 60 mL/min/1.73 m^2^ [20]. Patients were classified into five stages of CKD based on disease severity, according to the Kidney Disease Quality Outcome Initiative (K/DOQI) CKD classification. Stage 1: eGFR ≥ 90 mL/min/1.73 m^2^; Stage 2: eGFR 60–89 mL/min/1.73 m^2^; Stage 3: eGFR 30–59 mL/min/1.73 m^2^; Stage 4: eGFR 15–29 mL/min/1.73 m^2^; Stage 5: eGFR < 15 mL/min/1.73 m^2^.

### 2.3 Definition of Obesity‐Related Indices

Obesity‐related indices in current were calculated by the arithmetic formulas according to a previous study [17].

### 2.4 Assessment of Covariates

In examining the correlation between obesity‐related indices and DN, we developed multivariable‐adjusted models to isolate the influence of confounding variables on this relationship. The following covariates were included in this study: gender, age, race, education, marital status, family economic status (as measured by the Poverty Income Ratio (PIR)), smoking behaviour, and history of a range of important chronic diseases, including hypertension, coronary artery disease (CAD), stroke, and cancer. For racial categorisation, participants were subdivided into the following categories: Mexican American, non‐Hispanic white, non‐Hispanic black, and other racial group. The sample was divided into three categories based on the years of education completed: less than 9th grade, 9th through 12th grade, and more than 12th grade. To categorise family economic status, income was carefully divided into three intervals based on the PIR criterion, as officially defined by the U.S. government. For this study, smoking habits were assessed using standardised methods. Smoking status was defined by the number of cigarettes smoked by the participant, with a minimum of 100 required for classification as a current smoker. Chronic medical history was collected for HBP (whether the participant had ever been told they had high blood pressure or were currently taking prescription medication for high blood pressure), CAD (whether a doctor had ever told the participant that they had CAD, angina, or a heart attack), stroke (whether a doctor had ever told the participant that they had a stroke), and cancer (whether a doctor had ever told the participant that they had cancer).

### 2.5 Statistical Analysis

The test results selected the mean ± standard deviation or median (25th and 75th percentile) to characterise the variables. One‐way analysis of variance (ANOVA) or Kruskal–Wallis nonparametric tests were employed to ascertain differences between groups about the distribution characteristics of the variables in question. For categorical variables, the data were presented in the form of frequencies and percentages, and the chi‐square test was employed to analyse the differences between groups.

Multivariable logistic regression models were used to examine the relationship between the obesity‐related indices and DN with odds ratio (ORs) and 95% confidence intervals (CIs). Model 1 was unadjusted, and Model 2 was adjusted for age categorical, gender, race, education level, the ratio of family income to poverty, and BMI. Model 3 was further adjusted for age categorical, gender, race, education level, the ratio of family income to poverty, BMI, FBG, HBA1C, TG, HDL‐C, CAD, stroke, HBP, cancer, and smoke.

The employment of smooth curve fitting and generalised additive models allowed for the examination of whether the independent variable was segmented into distinct intervals, thereby assessing the non‐linear association between the independent variable and GDM. Further investigation of the diagnostic effectiveness of the METS‐IR using a receiver operating characteristic (ROC) curve was conducted. Empower(R) (X&Y Solutions Inc., MA, USA) and Stata (version 14.0) were used for statistical analysis. Statistical significance was set at *p*‐value < 0.05.

## 3 Results

### 3.1 Baseline Characteristics of Patients With DN

This study included 1718 patients with DM, among which 843 patients had DN, and 10,042 in the normal group. Baseline characterisation revealed significant differences between the two groups concerning several demographic and clinical variables. In particular, the mean age was significantly higher in the DN group (69.33 years) than in the DM group (55.00 years) and the normal group (46.32 years). The DN group comprised more males (57.17%). The racial distribution revealed that most individuals in the DN group were Non‐Hispanic White participants (66.51%). Additionally, significant differences were observed in educational level, with a higher proportion of individuals with higher education in the DN (50.52%) and DM groups (53.80%). The DM and DN groups exhibited a lower mean family income. Compared with the DM group, the prevalence of CAD, stroke, HBP, and HC was higher in the DN group, while the prevalence of smoking was lower in the DN group. Regarding the clinical parameters, the mean BMI, FBG, HbA1c, UCr, TG, WC, WHtR, BRI, VAI, LAP, and CMI were lower in the DN group than in the DM group (*p* < 0.05). In contrast, the mean urinary protein, HDL‐C, WWI, and ABSI were higher in the DN group than in the DM group (*p* < 0.05). It is noteworthy that the WHtR, BRI, VAI, LAP, and CMI were lower in the DN group than in the DM group (*p* < 0.05) while WWI and ABSI were higher in the DN group (*p* < 0.05), indicating an association between obesity‐related indices and DN in this cohort (Table 1).

**TABLE 1:** Baseline characteristics of participants with DN and DM.

	Normol	DM	DN	*p*
Age (year)	46.32 (45.80, 46.83)	55.00 (54.19, 55.82)	69.33 (68.28, 70.38)	< 0.0001
*Age categorical*				< 0.0001
≤ 55	69.72 (68.19, 71.20)	47.75 (44.07, 51.46)	11.93 (8.86, 15.88)	
> 55	30.28 (28.80, 31.81)	52.25 (48.54, 55.93)	88.07 (84.12, 91.14)	
PIR	3.04 (2.96, 3.12)	2.84 (2.70, 2.98)	2.56 (2.41, 2.72)	< 0.0001
BMI	28.53 (28.31, 28.76)	34.07 (33.54, 34.60)	30.39 (29.85, 30.92)	< 0.0001
FBG (mg/dL)	99.09 (98.73, 99.45)	154.41 (150.76, 158.06)	150.32 (145.26, 155.38)	< 0.0001
HBA1C	5.40 (5.38, 5.41)	7.14 (7.03, 7.25)	7.08 (6.94, 7.22)	< 0.0001
Urinary protein (mg/L)	19.69 (17.30, 22.07)	27.46 (24.61, 30.30)	379.75 (274.27, 485.23)	< 0.0001
Ucr (mg/dL)	125.56 (122.94, 128.17)	127.37 (122.38, 132.37)	116.33 (108.95, 123.70)	0.0479
TG (mmol/L)	1.30 (1.28, 1.33)	1.87 (1.73, 2.01)	1.76 (1.62, 1.90)	< 0.0001
HDL‐C (mmol/L)	1.43 (1.42, 1.45)	1.23 (1.20, 1.26)	1.26 (1.23, 1.29)	< 0.0001
WC	97.92 (97.36, 98.48)	113.18 (111.90, 114.47)	107.10 (105.79, 108.40)	< 0.0001
WWI	10.89 (10.86, 10.92)	11.58 (11.53, 11.64)	11.70 (11.64, 11.76)	< 0.0001
WHtR	0.58 (0.58, 0.58)	0.67 (0.67, 0.68)	0.64 (0.64, 0.65)	< 0.0001
ABSI	0.0812 (0.0810, 0.0813)	0.0836 (0.0834, 0.0839)	0.0857 (0.0853, 0.0861)	< 0.0001
BRI	5.17 (5.09, 5.25)	7.41 (7.22, 7.60)	6.59 (6.39, 6.78)	< 0.0001
VAI	1.78 (1.73, 1.83)	3.14 (2.83, 3.46)	2.77 (2.49, 3.04)	< 0.0001
LAP	50.84 (49.30, 52.38)	97.59 (90.20, 104.98)	82.42 (74.64, 90.20)	< 0.0001
CMI	0.65 (0.63, 0.67)	1.24 (1.12, 1.36)	1.10 (0.97, 1.23)	< 0.0001
*Gender*				0.0003
Male	47.25 (46.20, 48.30)	50.63 (46.98, 54.27)	57.17 (52.54, 61.68)	
Female	52.75 (51.70, 53.80)	49.37 (45.73, 53.02)	42.83 (38.32, 47.46)	
*Race*				< 0.0001
Mexican American	7.74 (6.40, 9.32)	10.69 (8.66, 13.13)	5.35 (3.66, 7.77)	
Other hispanic	5.81 (4.83, 6.97)	6.87 (5.43, 8.64)	4.22 (3.01, 5.87)	
Non‐hispanic white	68.16 (65.36, 70.82)	59.97 (55.60, 64.18)	66.51 (62.27, 70.50)	
Non‐hispanic black	10.43 (9.04, 12.01)	13.50 (11.11, 16.32)	15.52 (12.74, 18.77)	
Other race	7.87 (6.95, 8.90)	8.97 (7.23, 11.08)	8.40 (6.50, 10.80)	
*Education*				< 0.0001
Less than high school	13.65 (12.25, 15.17)	22.70 (19.94, 25.71)	21.66 (18.67, 24.97)	
High school or GED	21.61 (20.08, 23.22)	23.50 (20.39, 26.92)	27.83 (23.81, 32.24)	
Above high school	64.74 (62.32, 67.09)	53.80 (49.71, 57.85)	50.52 (45.91, 55.12)	
*CAD*				< 0.0001
NO	97.40 (96.90, 97.83)	92.99 (90.19, 95.04)	83.34 (80.24, 86.04)	
YES	2.60 (2.17, 3.10)	7.01 (4.96, 9.81)	16.66 (13.96, 19.76)	
*Stroke*				< 0.0001
NO	97.75 (97.33, 98.11)	95.37 (93.89, 96.51)	86.91 (83.21, 89.90)	
YES	2.25 (1.89, 2.67)	4.63 (3.49, 6.11)	13.09 (10.10, 16.79)	
*HBP*				< 0.0001
NO	71.04 (69.51, 72.52)	40.29 (36.39, 44.31)	23.65 (19.74, 28.07)	
YES	28.96 (27.48, 30.49)	59.71 (55.69, 63.61)	76.35 (71.93, 80.26)	
*HC*				< 0.0001
NO	68.58 (67.24, 69.88)	42.32 (38.88, 45.84)	37.28 (32.64, 42.17)	
YES	31.42 (30.12, 32.76)	57.68 (54.16, 61.12)	62.72 (57.83, 67.36)	
*Smoke*				0.0001
NO	57.30 (55.64, 58.94)	50.49 (46.87, 54.11)	51.00 (45.43, 56.56)	
YES	42.70 (41.06, 44.36)	49.51 (45.89, 53.13)	49.00 (43.44, 54.57)	

### 3.2 Areas Under the ROC Curve Analysis

**FIGURE 2:**
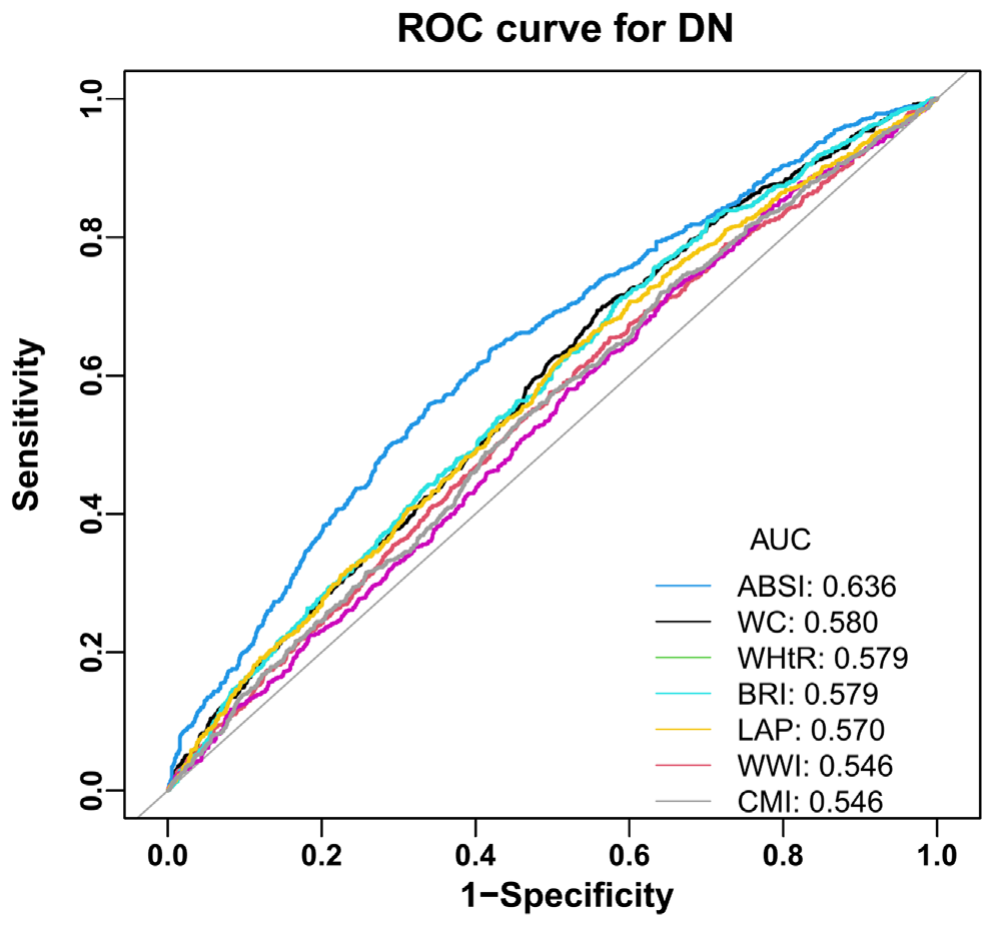
Diagnostic efficacy of obesity‐related indices for DN.

Finally, we compared the area under the ROC curves of DN with those of the variables that represent obesity‐related indices. The findings indicated that the area under the ROC curve for DN of ABSI was 0.636 (95% CI: 0.6136–0.6592, *p* < 0.05) (Figure 2) (Table S1). Moreover, differential analysis of ROC showed that ABSI was higher than BRI, WWI, and WHtR, whereas the difference between ABSI, BRI, WWI, WHtR, and DN was statistically significant (*p* < 0.05) (Figure 2).

### 3.3 Baseline Characteristics of the Study Population Stratified by Tertiles of ABSI, BRI and WWI Value

Thus, the study population was subsequently divided into four groups by the 100 times ABSI quartiles for better comparison of differences between groups, including Q1 (6.63, 8.12), Q2 (8.12, 8.42), Q3 (8.42, 8.72), Q4 (8.72, 9.86) (Table 2). As the quartiles of ABSI increased, the age, UACR, SCR, CAD (%), stroke (%), HBP (%), HC (%) and smoking (%) showed a gradual increase. On the contrary, as the quartiles of ABSI increased, the PIR, eGFR, BMI, and race (%) showed a gradual decrease. Interestingly, the level of FBG, HBA1C, and TG decreased as ABSI increased, while at Q4 (8.72, 9.86), the level of participants was higher than that of Q2 (8.12, 8.42) and Q3 (8.42, 8.72). On the contrary, the level of urine protein, Ucr, and HDL‐C increased as ABSI increased, while at Q4 (8.72, 9.86), the level of participants was lower than that of Q2 (8.12, 8.42) and Q3 (8.42, 8.72) (Table 2).

**TABLE 2:** Baseline characteristics of the study population stratified by tertiles of ABSI value.

	Q1	Q2	Q3	Q4	*p*
Age	52.76 (51.20, 54.32)	55.84 (54.78, 56.89)	61.51 (60.24, 62.78)	65.64 (64.54, 66.74)	< 0.0001
*Age categorical*					< 0.0001
≤ 55	58.20 (52.56, 63.63)	48.25 (43.48, 53.04)	29.11 (23.86, 35.00)	15.85 (12.26, 20.24)	
> 55	41.80 (36.37, 47.44)	51.75 (46.96, 56.52)	70.89 (65.00, 76.14)	84.15 (79.76, 87.74)	
PIR	2.58 (2.40, 2.76)	2.94 (2.75, 3.14)	2.82 (2.60, 3.03)	2.67 (2.50, 2.85)	0.0256
FBG (mg/dL)	152.19 (146.97, 157.41)	158.73 (151.47, 165.99)	147.45 (142.62, 152.28)	154.94 (147.57, 162.31)	0.0498
HBA1C	7.11 (6.94, 7.27)	7.29 (7.09, 7.49)	6.98 (6.84, 7.12)	7.12 (6.92, 7.33)	0.0621
Urine protein (mg/L)	162.29 (67.72,256.86)	103.21 (43.45,162.98)	121.57 (75.37,167.76)	120.01 (79.14,160.88)	0.7908
Ucr (mg/dL)	124.93 (118.08, 131.78)	125.51 (116.76, 134.25)	131.61 (121.28, 141.94)	115.73 (109.96, 121.50)	0.0639
UACR	148.77 (73.84, 223.70)	90.08 (48.60, 131.56)	104.83 (64.84, 144.81)	133.24 (81.44, 185.03)	0.3782
eGFR	95.99 (92.43, 99.55)	90.86 (86.99, 94.73)	78.34 (74.28, 82.40)	73.86 (69.80, 77.93)	< 0.0001
Scr	0.88 (0.85, 0.92)	0.94 (0.85, 1.03)	0.97 (0.90, 1.04)	0.99 (0.94, 1.04)	0.0074
TG (mmol/L)	1.86 (1.56, 2.16)	1.94 (1.77, 2.10)	1.69 (1.57, 1.82)	1.83 (1.69, 1.97)	0.1360
HDL‐C (mmol/L)	1.28 (1.25, 1.31)	1.23 (1.20, 1.26)	1.24 (1.18, 1.31)	1.21 (1.17, 1.25)	0.0486
BMI	35.50 (34.49, 36.52)	33.06 (32.23, 33.90)	32.14 (31.32, 32.96)	31.50 (30.85, 32.14)	< 0.0001
*Gender*					< 0.0001
Male	40.23 (34.86, 45.84)	56.11 (50.51, 61.55)	58.86 (52.99, 64.49)	55.21 (49.31, 60.96)	
Female	59.77 (54.16, 65.14)	43.89 (38.45, 49.49)	41.14 (35.51, 47.01)	44.79 (39.04, 50.69)	
*Race*					< 0.0001
Mexican American	12.69 (9.70, 16.43)	10.10 (7.70, 13.15)	9.09 (6.73, 12.17)	4.94 (3.45, 7.02)	
Other hispanic	7.03 (5.41, 9.08)	7.18 (5.00, 10.22)	6.32 (4.27, 9.26)	4.15 (2.92, 5.87)	
Non‐hispanic white	47.79 (41.57, 54.09)	57.74 (51.61, 63.64)	64.73 (57.90, 71.01)	76.46 (72.10, 80.32)	
Non‐hispanic black	22.91 (18.83, 27.57)	15.13 (11.83, 19.15)	10.94 (8.03, 14.73)	7.60 (5.82, 9.88)	
Other race	9.58 (7.03, 12.91)	9.84 (7.44, 12.91)	8.92 (6.13, 12.81)	6.85 (5.15, 9.04)	
*Education level*					0.1766
Less than high school	21.99 (18.39, 26.08)	20.62 (16.90, 24.92)	23.55 (19.08, 28.69)	23.10 (19.08, 27.68)	
High school or GED	25.09 (21.06, 29.59)	24.17 (20.01, 28.89)	19.99 (15.57, 25.29)	28.59 (23.28, 34.58)	
Above high school	52.92 (47.72, 58.07)	55.21 (49.83, 60.47)	56.46 (50.97, 61.80)	48.30 (41.40, 55.27)	
*CAD*					< 0.0001
NO	96.78 (94.55, 98.12)	90.50 (86.36, 93.47)	89.83 (83.51, 93.90)	84.04 (79.79, 87.54)	
YES	3.22 (1.88, 5.45)	9.50 (6.53, 13.64)	10.17 (6.10, 16.49)	15.96 (12.46, 20.21)	
*Stroke*					0.0735
NO	94.88 (92.05, 96.74)	93.99 (90.92, 96.06)	92.95 (89.79, 95.19)	90.31 (87.16, 92.75)	
YES	5.12 (3.26, 7.95)	6.01 (3.94, 9.08)	7.05 (4.81, 10.21)	9.69 (7.25, 12.84)	
*HBP*					0.0099
NO	41.03 (34.62, 47.76)	40.07 (34.23, 46.21)	32.42 (26.12, 39.44)	29.13 (24.52, 34.22)	
YES	58.97 (52.24, 65.38)	59.93 (53.79, 65.77)	67.58 (60.56, 73.88)	70.87 (65.78, 75.48)	
*HC*					0.0087
NO	47.62 (43.01, 52.28)	42.50 (36.86, 48.34)	38.36 (32.91, 44.11)	35.46 (30.39, 40.88)	
YES	52.38 (47.72, 56.99)	57.50 (51.66, 63.14)	61.64 (55.89, 67.09)	64.54 (59.12, 69.61)	
*Smoke*					< 0.0001
NO	63.95 (59.16, 68.49)	51.87 (44.81, 58.86)	45.43 (40.01, 50.96)	42.05 (36.28, 48.04)	
YES	36.05 (31.51, 40.84)	48.13 (41.14, 55.19)	54.57 (49.04, 59.99)	57.95 (51.96, 63.72)	

Thus, the study population was subsequently divided into four groups by the BRI quartiles for better comparison of differences between groups, including Q1 (1.49, 5.15), Q2 (5.15, 6.57), Q3 (6.57, 8.19), Q4 (8.20, 18.99) (Table 3). As the quartiles of BRI increased, the eGFR, BMI, percent of non‐Hispanic White (%) and HBP (%) showed a gradual increased. On the contrary, as the quartiles of BRI increased, the SCR, HDL‐C and percent of male patients showed a gradual decreased (Table 3). the level of TG increased as ABSI increased, while at Q4 (8.20, 18.99), the level of participates was lower than that of Q2 (5.15, 6.57), Q3 (6.57, 8.19) (Table 3).

**TABLE 3:** Baseline characteristics of the study population stratified by tertiles of BRI value.

	Q1	Q2	Q3	Q4	*p*
Age	58.98 (57.40, 60.56)	60.87 (59.42, 62.33)	60.23 (58.93, 61.54)	56.30 (54.94, 57.67)	< 0.0001
*Age categorical*					0.0410
≤ 55	40.22 (34.78, 45.92)	34.42 (28.57, 40.77)	33.00 (28.21, 38.16)	42.47 (36.91, 48.24)	
> 55	59.78 (54.08, 65.22)	65.58 (59.23, 71.43)	67.00 (61.84, 71.79)	57.53 (51.76, 63.09)	
PIR	2.80 (2.61, 2.98)	2.94 (2.74, 3.15)	2.61 (2.41, 2.80)	2.67 (2.48, 2.86)	0.0942
FBG (mg/dL)	151.93 (144.92, 158.94)	153.11 (147.56, 158.66)	154.87 (148.66, 161.07)	153.19 (147.04, 159.34)	0.9464
HBA1C	7.05 (6.85, 7.24)	7.04 (6.89, 7.20)	7.16 (6.99, 7.33)	7.22 (7.04, 7.41)	0.4396
Urine protein (mg/L)	99.57 (64.34, 134.80)	91.16 (64.27, 118.05)	208.50 (104.38, 312.63)	105.44 (64.98, 145.90)	0.1992
Ucr (mg/dL)	119.45 (110.92, 127.99)	126.35 (115.04, 137.67)	127.60 (119.10, 136.09)	123.37 (116.46, 130.29)	0.5226
UACR	107.95 (67.81, 148.09)	95.50 (58.87, 132.13)	167.25 (93.00, 241.49)	106.41 (61.65, 151.16)	0.4279
eGFR	71.98 (69.16, 74.80)	77.99 (74.75, 81.23)	83.14 (79.75, 86.54)	101.60 (96.99, 106.20)	< 0.0001
Scr	1.02 (0.92, 1.12)	0.95 (0.90, 1.00)	0.95 (0.90, 0.99)	0.89 (0.85, 0.92)	0.0227
TG (mmol/L)	1.52 (1.26, 1.77)	1.89 (1.70, 2.08)	1.98 (1.75, 2.21)	1.88 (1.74, 2.02)	0.0338
HDL‐C (mmol/L)	1.34 (1.30, 1.38)	1.26 (1.19, 1.33)	1.20 (1.16, 1.23)	1.18 (1.15, 1.22)	< 0.0001
BMI	24.97 (24.66, 25.28)	29.67 (29.36, 29.97)	33.63 (33.31, 33.95)	41.70 (41.04, 42.36)	< 0.0001
*Gender*					< 0.0001
Male	68.65 (62.88, 73.90)	60.79 (55.59, 65.76)	48.07 (42.82, 53.37)	37.15 (31.32, 43.38)	
Female	31.35 (26.10, 37.12)	39.21 (34.24, 44.41)	51.93 (46.63, 57.18)	62.85 (56.62, 68.68)	
*Race*					< 0.0001
Mexican American	7.30 (5.29, 10.01)	9.92 (7.21, 13.50)	9.89 (7.15, 13.53)	9.21 (6.88, 12.23)	
Other hispanic	6.75 (4.96, 9.14)	5.64 (3.84, 8.21)	6.22 (4.65, 8.28)	6.07 (4.08, 8.95)	
Non‐hispanic white	53.28 (47.70, 58.78)	62.80 (56.36, 68.81)	64.06 (58.11, 69.60)	65.63 (60.12, 70.76)	
Non‐hispanic black	14.85 (11.88, 18.41)	13.55 (10.52, 17.28)	13.73 (10.85, 17.23)	14.21 (10.95, 18.24)	
Other race	17.81 (14.39, 21.84)	8.10 (6.02, 10.82)	6.10 (3.64, 10.04)	4.88 (3.13, 7.51)	
*Education level*					0.2135
Less than high school	19.88 (16.21, 24.14)	22.21 (17.95, 27.14)	23.39 (19.41, 27.92)	23.36 (19.47, 27.74)	
High school or GED	27.69 (23.22, 32.66)	21.73 (17.34, 26.88)	21.30 (17.11, 26.19)	27.45 (22.48, 33.05)	
Above high school	52.42 (46.24, 58.53)	56.06 (50.10, 61.85)	55.30 (49.15, 61.30)	49.20 (43.32, 55.10)	
*CAD*					0.1903
NO	92.40 (88.75, 94.94)	87.24 (81.25, 91.52)	90.10 (86.76, 92.67)	91.31 (87.73, 93.92)	
YES	7.60 (5.06, 11.25)	12.76 (8.48, 18.75)	9.90 (7.33, 13.24)	8.69 (6.08, 12.27)	
*Stroke*					0.5743
NO	93.25 (89.43, 95.76)	94.14 (91.15, 96.16)	93.18 (90.94, 94.89)	91.61 (88.14, 94.13)	
YES	6.75 (4.24, 10.57)	5.86 (3.84, 8.85)	6.82 (5.11, 9.06)	8.39 (5.87, 11.86)	
*HBP*					< 0.0001
NO	46.96 (40.68, 53.35)	37.89 (32.22, 43.92)	30.65 (25.72, 36.07)	29.21 (24.58, 34.32)	
YES	53.04 (46.65, 59.32)	62.11 (56.08, 67.78)	69.35 (63.93, 74.28)	70.79 (65.68, 75.42)	
*HC*					0.3131
NO	43.82 (37.98, 49.83)	36.37 (31.04, 42.05)	42.64 (36.57, 48.93)	41.22 (35.95, 46.70)	
YES	56.18 (50.17, 62.02)	63.63 (57.95, 68.96)	57.36 (51.07, 63.43)	58.78 (53.30, 64.05)	
*Smoke*					0.1707
NO	50.92 (45.06, 56.76)	48.95 (43.68, 54.25)	55.98 (49.50, 62.26)	47.35 (41.31, 53.48)	
YES	49.08 (43.24, 54.94)	51.05 (45.75, 56.32)	44.02 (37.74, 50.50)	52.65 (46.52, 58.69)	

Thus, the study population was subsequently divided into four groups by the 10 times WWI quartiles for better comparison of differences between groups, including Q1 (8.85, 11.12), Q2 (11.12, 11.61), Q3 (11.61, 12.08), Q4 (12.08, 14.20) (Table 4). As the quartiles of WWI increased, the eGFR, BMI, percent of non‐Hispanic White (%) and HBP (%) showed a gradual increased. On the contrary, as the quartiles of BRI increased, the SCR, HDL‐C and percent of male patients showed a gradual decreased (Table 4). the level of TG increased as ABSI increased, while at Q4 (12.08, 14.20), the level of participates was lower than that of Q2 (11.12, 11.61), Q3 (11.61, 12.08) (Table 4).

**TABLE 4:** Baseline characteristics of the study population stratified by tertiles of WWI value.

	Q1	Q2	Q3	Q4	*p*
Age	58.98 (57.40, 60.56)	60.87 (59.42, 62.33)	60.23 (58.93, 61.54)	56.30 (54.94, 57.67)	< 0.0001
*AGE categorical*					0.0410
≤ 55	40.22 (34.78, 45.92)	34.42 (28.57, 40.77)	33.00 (28.21, 38.16)	42.47 (36.91, 48.24)	
> 55	59.78 (54.08, 65.22)	65.58 (59.23, 71.43)	67.00 (61.84, 71.79)	57.53 (51.76, 63.09)	
PIR	2.80 (2.61, 2.98)	2.94 (2.74, 3.15)	2.61 (2.41, 2.80)	2.67 (2.48, 2.86)	0.0942
FBG (mg/dL)	151.93 (144.92, 158.94)	153.11 (147.56, 158.66)	154.87 (148.66, 161.07)	153.19 (147.04, 159.34)	0.9464
HBA1C	7.05 (6.85, 7.24)	7.04 (6.89, 7.20)	7.16 (6.99, 7.33)	7.22 (7.04, 7.41)	0.4396
Urine protein (mg/L)	99.57 (64.34, 134.80)	91.16 (64.27, 118.05)	208.50 (104.38, 312.63)	105.44 (64.98, 145.90)	0.1992
Ucr (mg/dL)	119.45 (110.92, 127.99)	126.35 (115.04, 137.67)	127.60 (119.10, 136.09)	123.37 (116.46, 130.29)	0.5226
UACR	107.95 (67.81, 148.09)	95.50 (58.87, 132.13)	167.25 (93.00, 241.49)	106.41 (61.65, 151.16)	0.4279
eGFR	71.98 (69.16, 74.80)	77.99 (74.75, 81.23)	83.14 (79.75, 86.54)	101.60 (96.99, 106.20)	< 0.0001
SCR	1.02 (0.92, 1.12)	0.95 (0.90, 1.00)	0.95 (0.90, 0.99)	0.89 (0.85, 0.92)	0.0227
TG (mmol/L)	1.52 (1.26, 1.77)	1.89 (1.70, 2.08)	1.98 (1.75, 2.21)	1.88 (1.74, 2.02)	0.0338
HDL‐C (mmol/L)	1.34 (1.30, 1.38)	1.26 (1.19, 1.33)	1.20 (1.16, 1.23)	1.18 (1.15, 1.22)	< 0.0001
BMI	24.97 (24.66, 25.28)	29.67 (29.36, 29.97)	33.63 (33.31, 33.95)	41.70 (41.04, 42.36)	< 0.0001
*Gender*					< 0.0001
Male	68.65 (62.88, 73.90)	60.79 (55.59, 65.76)	48.07 (42.82, 53.37)	37.15 (31.32, 43.38)	
Female	31.35 (26.10, 37.12)	39.21 (34.24, 44.41)	51.93 (46.63, 57.18)	62.85 (56.62, 68.68)	
*Race*					< 0.0001
Mexican American	7.30 (5.29, 10.01)	9.92 (7.21, 13.50)	9.89 (7.15, 13.53)	9.21 (6.88, 12.23)	
Other hispanic	6.75 (4.96, 9.14)	5.64 (3.84, 8.21)	6.22 (4.65, 8.28)	6.07 (4.08, 8.95)	
Non‐hispanic white	53.28 (47.70, 58.78)	62.80 (56.36, 68.81)	64.06 (58.11, 69.60)	65.63 (60.12, 70.76)	
Non‐hispanic black	14.85 (11.88, 18.41)	13.55 (10.52, 17.28)	13.73 (10.85, 17.23)	14.21 (10.95, 18.24)	
Other race	17.81 (14.39, 21.84)	8.10 (6.02, 10.82)	6.10 (3.64, 10.04)	4.88 (3.13, 7.51)	
*Education level*					0.2135
Less than high school	19.88 (16.21, 24.14)	22.21 (17.95, 27.14)	23.39 (19.41, 27.92)	23.36 (19.47, 27.74)	
High school or GED	27.69 (23.22, 32.66)	21.73 (17.34, 26.88)	21.30 (17.11, 26.19)	27.45 (22.48, 33.05)	
Above high school	52.42 (46.24, 58.53)	56.06 (50.10, 61.85)	55.30 (49.15, 61.30)	49.20 (43.32, 55.10)	
*CAD*					0.1903
NO	92.40 (88.75, 94.94)	87.24 (81.25, 91.52)	90.10 (86.76, 92.67)	91.31 (87.73, 93.92)	
YES	7.60 (5.06, 11.25)	12.76 (8.48, 18.75)	9.90 (7.33, 13.24)	8.69 (6.08, 12.27)	
*Stroke*					0.5743
NO	93.25 (89.43, 95.76)	94.14 (91.15, 96.16)	93.18 (90.94, 94.89)	91.61 (88.14, 94.13)	
YES	6.75 (4.24, 10.57)	5.86 (3.84, 8.85)	6.82 (5.11, 9.06)	8.39 (5.87, 11.86)	
*HBP*					< 0.0001
NO	46.96 (40.68, 53.35)	37.89 (32.22, 43.92)	30.65 (25.72, 36.07)	29.21 (24.58, 34.32)	
YES	53.04 (46.65, 59.32)	62.11 (56.08, 67.78)	69.35 (63.93, 74.28)	70.79 (65.68, 75.42)	
*HC*					0.3131
NO	43.82 (37.98, 49.83)	36.37 (31.04, 42.05)	42.64 (36.57, 48.93)	41.22 (35.95, 46.70)	
YES	56.18 (50.17, 62.02)	63.63 (57.95, 68.96)	57.36 (51.07, 63.43)	58.78 (53.30, 64.05)	
*Smoke*					0.1707
NO	50.92 (45.06, 56.76)	48.95 (43.68, 54.25)	55.98 (49.50, 62.26)	47.35 (41.31, 53.48)	
YES	49.08 (43.24, 54.94)	51.05 (45.75, 56.32)	44.02 (37.74, 50.50)	52.65 (46.52, 58.69)	

### 3.4 The Association Between Obesity‐Related Indices and DN

**FIGURE 3:**
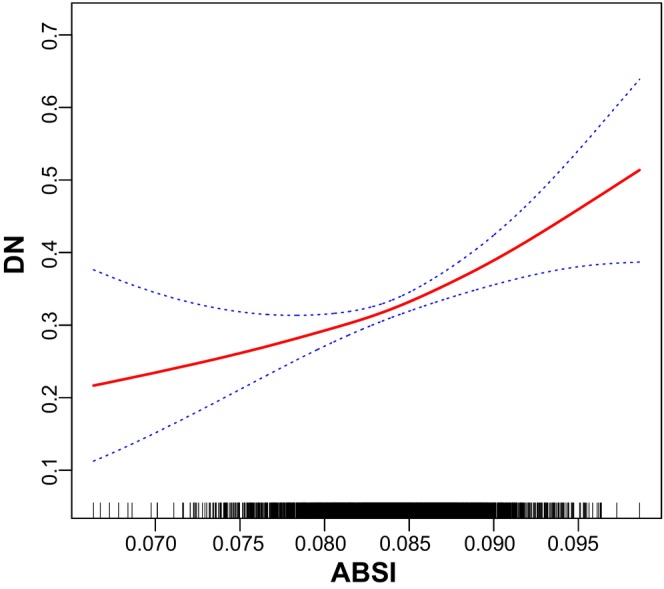
Smooth curve fitting for the relations between 100 times ABSI with DN.

**FIGURE 4:**
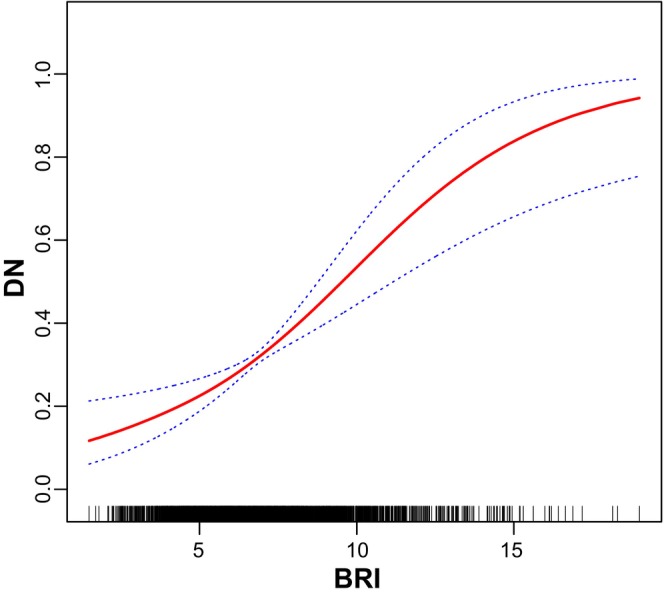
Smooth curve fitting for the relations between BRI and DN.

**FIGURE 5:**
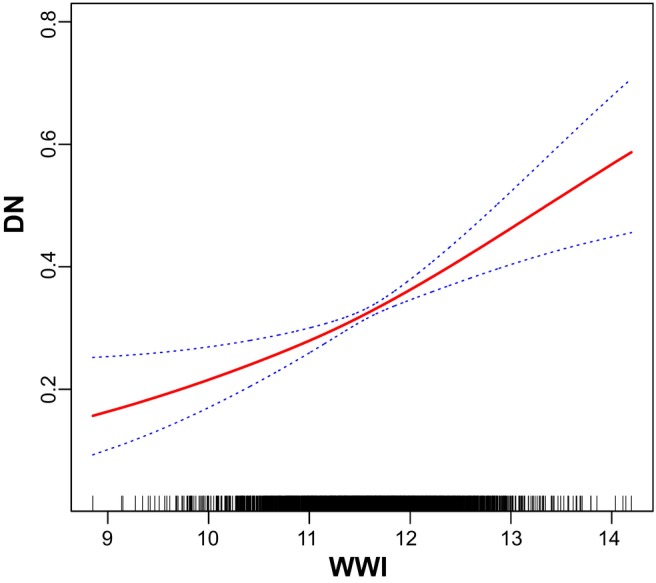
Smooth curve fitting for the relations between WWI and DN.

**FIGURE 6:**
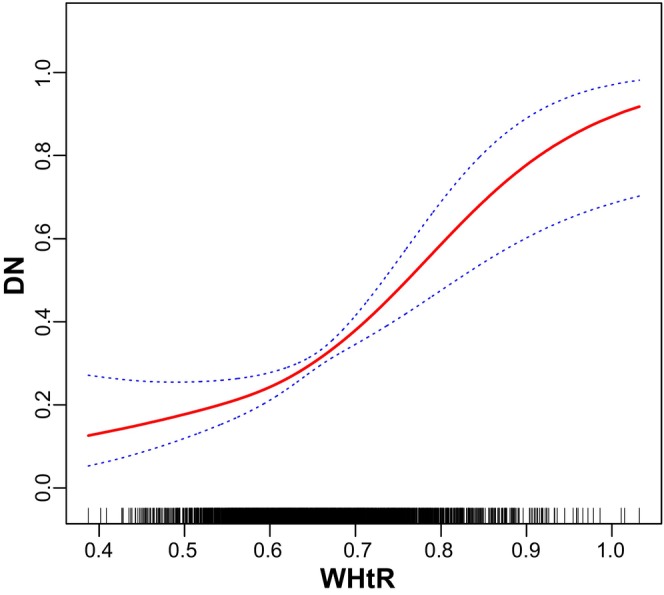
Smooth curve fitting for the relations between WHtR and DN.

DN was defined as the dependent variable, and the above‐mentioned relevant indicators (Tables 1, 2, 3, 4) as independent variables. Following the adjustment for potential confounders including adjusted for age categorical, gender, race, education level, the ratio of family income to poverty, BMI, FBG, HBA1C, TG, HDL‐C, CAD, Stroke, HBP, HC and Smoke, multivariable logistic regression model 3 revealed OR (95% CI) of 0.81, 1.12 and 1.17 for participants in the Q2, Q3 and Q4 tertiles of ABSI, respectively, although without statistical significance (Table 5). However, smooth curve fitting for the relationship between ABSI and DN was nonlinear and U‐shaped (*p* < 0.001; Figure 3). Multivariable logistic regression model 3 revealed OR (95% CI) of 0.80, 1.60 and 1.80 for participants in the Q2, Q3 and Q4 tertiles of BRI, respectively (Table 6). Smooth curve fitting for the relationship between BRI and DN was nonlinear and U‐shaped (*p* < 0.001; Figure 4). Multivariable logistic regression model 3 revealed OR (95% CI) of 1.17, 1.55 and 1.63 for participants in the Q2, Q3 and Q4 tertiles of WWI, respectively (Table 7). Smooth curve fitting for the relationship between WWI and DN was nonlinear and U‐shaped (*p* < 0.001; Figure 5). In addition, multivariable logistic regression model 3 revealed OR (95% CI) of 0.80, 1.60 and 1.80 for participants in the Q2, Q3 and Q4 tertiles of 10 times WHtR, respectively (Table 8). Smooth curve fitting for the relationship between WHtR and DN was nonlinear and U‐shaped (*p* < 0.001; Figure 6).

**TABLE 5:** Association between 100 times ABSI with DN, weighted.

	DN OR (95% CI), *p*					
	100 times ABSI					
	Per1 increment	Q1 (6.63, 8.12)	Q2 (8.12, 8.42)	Q3 (8.42, 8.72)	Q4 (8.72, 9.86)	P for trend
Model 1	3.00 (2.31, 3.88), < 0.0001	Ref.	1.12 (0.77, 1.61), 0.5581	2.01 (1.43, 2.85), 0.0001	2.78 (2.03, 3.80), < 0.0001	1.45 (1.31, 1.61), < 0.0001
Model 2	1.46 (1.09, 1.94), 0.0119	Ref.	0.85 (0.55, 1.31), 0.4703	1.18 (0.78, 1.77), 0.4442	1.25 (0.87, 1.80), 0.2345	1.11 (0.99, 1.25), 0.0756
Model 3	1.39 (1.03, 1.89), 0.0368	Ref.	0.81 (0.53, 1.26), 0.3564	1.12 (0.73, 1.71), 0.6087	1.17 (0.81, 1.69), 0.3977	1.09 (0.97, 1.23), 0.1523

*Note:* Model 1: unadjusted. Model 2: adjusted for age categorical, gender, race, education level, the ratio of family income to poverty, and BMI. Model 3: adjusted for age categorical, gender, race, education level, the ratio of family income to poverty, BMI, FBG, HBA1C, TG, HDL‐C, CAD, Stroke, HBP, HC, Smoke.

Abbreviations: 95% CI, 95% confidence interval; OR, odds ratio.

**TABLE 6:** Association between BRI with DN, weighted.

	DN OR (95% CI), *p*					
	BRI					
	Per1 increment	Q1 (1.49, 5.15)	Q2 (5.15, 6.57)	Q3 (6.57, 8.19)	Q4 (8.20, 18.99)	P for trend
Model 1	0.87 (0.83, 0.91), < 0.0001	Ref.	0.61 (0.45, 0.82), 0.0017	0.77 (0.56, 1.07), 0.1189	0.37 (0.27, 0.51), < 0.0001	0.77 (0.69, 0.85), < 0.0001
Model 2	1.28 (1.11, 1.47), 0.0011	Ref.	0.86 (0.60, 1.22), 0.3954	1.68 (1.00, 2.83), 0.0534	1.85 (0.86, 3.98), 0.1204	1.31 (1.02, 1.67), 0.0354
Model 3	1.26 (1.09, 1.47), 0.0033	Ref.	0.80 (0.55, 1.15), 0.2316	1.60 (0.93, 2.75), 0.0955	1.80 (0.84, 3.89), 0.1374	1.30 (1.01, 1.67), 0.0455

**TABLE 7:** Association between WWI and DN, weighted.

	DN OR (95% CI), *p*					
	WWI					
	Per1 increment	Q1 (8.85, 11.12)	Q2 (11.12, 11.61)	Q3 (11.61, 12.08)	Q4 (12.08, 14.20)	P for trend
Model 1	1.25 (1.09, 1.45), 0.0024	Ref.	1.19 (0.87, 1.63), 0.2681	1.45 (1.08, 1.96), 0.0168	1.41 (1.02, 1.95), 0.0424	1.13 (1.02, 1.24), 0.0191
Model 2	1.50 (1.23, 1.83), 0.0002	Ref.	1.21 (0.82, 1.79), 0.3481	1.60 (1.09, 2.35), 0.0195	1.67 (1.08, 2.60), 0.0251	1.20 (1.04, 1.38), 0.0119
Model 3	1.46 (1.18, 1.81), 0.0009	Ref.	1.17 (0.77, 1.78), 0.4557	1.55 (1.02, 2.35), 0.0454	1.63 (1.02, 2.60), 0.0463	1.19 (1.03, 1.38), 0.0230

**TABLE 8:** Association between 10 times WHtR with DN, weighted.

	DN OR (95% CI), *p*					
	10 times WHtR					
	Per1 increment	Q1 (3.87, 5.88)	Q2 (5.88, 6.48)	Q3 (6.48, 7.11)	Q4 (7.11, 10.32)	P for trend
Model 1	0.03 (0.01, 0.10), < 0.0001	Ref.	0.61 (0.45, 0.82), 0.0017	0.77 (0.56, 1.07), 0.1189	0.37 (0.27, 0.51), < 0.0001	0.77 (0.69, 0.85), < 0.0001
Model 2	1.83 (1.28, 2.63), 0.0015	Ref.	0.86 (0.60, 1.22), 0.3954	1.68 (1.00, 2.83), 0.0534	1.85 (0.86, 3.98), 0.1204	1.31 (1.02, 1.67), 0.0354
Model 3	1.75 (1.19, 2.59), 0.0061	Ref.	0.80 (0.55, 1.15), 0.2316	1.60 (0.93, 2.75), 0.0955	1.80 (0.84, 3.89), 0.1374	1.30 (1.01, 1.67), 0.0455

### 3.5 Obesity‐Related Indices Levels of Different CKD Stages in Patients With DN

**FIGURE 7:**
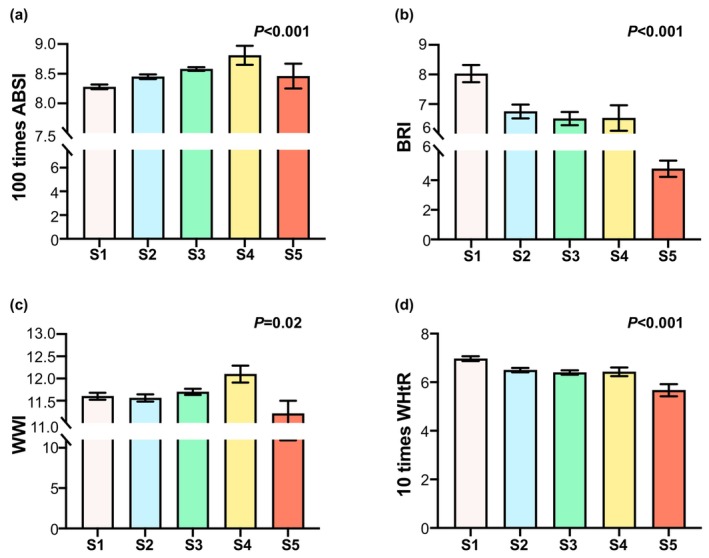
Obesity‐related indices levels of different CKD stages in patients with DN.

As the CKD stage increased, the values of obesity‐related indices demonstrated a differential trajectory. In particular, the value of BRI and WHtR exhibited a gradual decrease from Stage 1 to Stage 5. However, ABSI and WWI exhibited the same trend, gradually increasing from Stage 1 to Stage 4 and then decreasing at Stage 5 (Figure 7).

## 4 Discussion

Current study focused on which obesity‐related indices can better predict the occurrence of GDM using data from the nationally representative NHANES (2007–2018). A strong association was observed between higher ABSI, WWI, BRI, and WHtR and elevated likelihood of DN occurrence. The ABSI offers incremental value in model predictive power for DN in patients with DM than other obesity‐related indices. As renal function deteriorates, BRI and WHtR show a significant negative correlation with eGFR.

WC serves as an indicator of central obesity and is closely linked to abdominal fat, which is the primary contributor to metabolic disorders [21]. For an extended period, WC has been regarded as a more precise predictor of the prevalence and incidence of type 2 diabetes than BMI [22]. Nevertheless, due to its close correlation with BMI, WC's utility is somewhat limited beyond what BMI offers [23]. To address the issue of confounding resulting from the high correlation (~0.8) between BMI and WC, Krakauer et al. introduced ABSI, which adjusts WC for weight and height [23]. Similar to BMI, which adjusts weight for height, ABSI provides an alternative measure. In a study involving US adults with DM from the NHANES, ABSI demonstrated a linear and positive association with the risk of total cardiovascular disease mortality, especially among males and younger patients [24]. This research also identified ABSI as a reliable predictor of the onset of DN in DM patients. Yousung Park et al. proposed the WWI, which assesses obesity by normalising waist circumference to weight [25]. Their study revealed that WWI and ABSI were notably higher in DM patients who developed DN compared to those who did not. Moreover, among DN patients, WWI and ABSI exhibited a gradual increase as eGFR decreased across CKD stages S1–S4, but they significantly decreased in stage S5.

Previous studies have indicated that, apart from CKD or hypercholesterolemia, the BRI and WHR outperform other indices in predicting cardiovascular disease (CVD) risk factors [26]. Beyond overweight and obese males, BRI has consistently demonstrated its predictive capability for renal function in all CVD patients, a result well corroborated by other research [27]. BRI serves as a reliable indicator of visceral fat content [13]. Prior research has also shown that WHtR is the optimal metric for assessing renal function in overweight and obese male CVD patients. This study revealed that while BRI and WHtR may not be as effective as ABSI in predicting DN, they exhibit a significant positive correlation with eGFR in DN patients, thereby effectively predicting the progression of renal function in these patients. Notably, WHtR demonstrates a predictive ability comparable to that of BRI and is simpler to calculate. Therefore, this study suggests that WHtR is the preferred metric for predicting renal function in DN patients.

A hallmark characteristic of obesity is the accumulation of adipose tissue. Presently, adipose tissue has been the most extensively studied aspect in relation to DN, as it functions as a crucial endocrine organ that secretes diverse adipokines, acting on various target organs to maintain bodily homeostasis [28]. As a metabolic disorder, the role of adipokines in DN has garnered increasing attention [29]. On one hand, the dysregulation of adipose tissue during the progression of DN leads to abnormal secretion of adipokines; on the other hand, impaired renal function in DN results in the inability to excrete various adipokines through the kidneys. This study discovered that indicators related to adipose tissue distribution and metabolism, including BMI, WC, WHR, WHtR, LAP, BRI, VAI, ABSI, WWI, and CMI, show significant differences between DN patients and the normal population. Although the underlying mechanisms remain unclear, there is no doubt that the adipose–renal axis may emerge as a novel target for renal prevention and treatment of diabetic nephropathy in the future.

## 5 Conclusion

In summary, this study showed obesity‐related indices were closely related to the occurrence of DN, in particular ABSI, WWI, BRI and WHtR. The ABSI offered incremental value in model predictive power for DN in patients with DM than other obesity‐related indices. As renal function deteriorates, BRI and WHtR show a significant negative correlation with eGFR. Thus, ABSI, BRI and WHtR could be a convenient tool for clinicians to stratify DN patient risks and plan preventive and treatment strategies.

## 6 Strengths and Limitations of the Study

Our study boasted a substantial research population and an extended follow‐up period, providing us with a robust foundation to gain a deeper and more comprehensive understanding of the intricate relationship between obesity‐related indices and diabetic nephropathy (DN). This large‐scale and long‐term approach enabled us to capture more nuanced patterns and potential long‐term effects that might not be apparent in smaller or shorter studies. To delve even deeper into the data, we employed the restricted cubic spline (RCS) model. This sophisticated statistical tool allowed us to meticulously analyse the nonlinear relationship between the obesity‐related indices and DN. By doing so, we were able to uncover potential complex associations that go beyond simple linear correlations, offering a more accurate and detailed picture of how these indices interact with the development and progression of DN.

However, it is important to acknowledge the limitations of our study. One significant drawback is that we relied on self‐reported data for both DN diagnosis and living habits. This self‐reporting approach is susceptible to memory bias, as participants may not accurately recall or report their health status and daily habits. Such inaccuracies could potentially skew the results and affect the validity of our findings. Moreover, we only collected the baseline values of the obesity‐related indices. This means that we did not account for any changes in these indices that may have occurred during the course of diabetes mellitus (DM) and its progression to DN. As obesity is a dynamic condition that can change over time due to various factors such as lifestyle modifications, medical interventions, or the natural progression of the disease, the lack of longitudinal data on these indices limits our ability to fully understand their impact on DN development and progression.

## Author Contributions

Wei Zhang: conceptualisation, data curation, methodology, writing‐original draft preparation, data curation and methodology, software, methodology; Yao Liu: validation, visualisation and supervision, writing‐reviewing and editing, writing‐reviewing and editing.

## Ethics Statement

Before data from this study were included in the NHANES public database, all participants signed informed consent forms, adhering to the principles outlined in the Declaration of Helsinki, and were reviewed and approved by the NCHS Ethical Review Board.

## Conflicts of Interest

The authors declare no conflicts of interest.

## Supporting information


**Table S1:** ROC analysis for continuous predictor.

## Data Availability

The datasets generated and/or analysed during the current study are available in the [NHANES database] repository [http://www.cdc.gov/nchs/nhanes.htm].
