# Supplementary material for: The Obesity‐Related Indices Are Useful for Predicting Diabetic Nephropathy in Patients With Type II Diabetes Mellitus: A Retrospective Cohort Study of NHANES
Source: Endocrinol Diabetes Metab. 2025 Aug 8;8(5):e70087. doi: 10.1002/edm2.70087 (PMC12334795; doi:10.1002/edm2.70087)
Supplement: Supplementary file 1 — Table S1: ROC analysis for continuous predictor. [file EDM2-8-e70087-s001.docx]

**Supplementary Table 1. ROC analysis for continuous predictor**

| Test | ROC area(AUC) | 95%CI low | 95%CI upp | Specificity | Sensitivity |
| --- | --- | --- | --- | --- | --- |
| WC | 0.5805 | 0.5573 | 0.6036 | 0.4418 | 0.6940 |
| WWI | 0.5461 | 0.5224 | 0.5698 | 0.4249 | 0.6548 |
| WHTR | 0.5794 | 0.5562 | 0.6026 | 0.3673 | 0.7568 |
| ABSI | 0.6364 | 0.6136 | 0.6592 | 0.6612 | 0.5575 |
| BRI | 0.5794 | 0.5562 | 0.6026 | 0.3673 | 0.7568 |
| VAI | 0.5351 | 0.5115 | 0.5587 | 0.3434 | 0.7260 |
| LAP | 0.5695 | 0.5460 | 0.5930 | 0.4878 | 0.6263 |
| CMI | 0.5457 | 0.5221 | 0.5693 | 0.5431 | 0.5362 |
